# Numerical approach to investigate MR imaging artifacts from orthopedic implants at different field strengths according to ASTM F2119

**DOI:** 10.1007/s10334-023-01074-2

**Published:** 2023-03-18

**Authors:** Tobias Spronk, Oliver Kraff, Gregor Schaefers, Harald H. Quick

**Affiliations:** 1 Erwin L. Hahn Institute for MR Imaging, University of Duisburg-Essen, Kokereiallee 7, Building C84, 45141 Essen, Germany; 2 High-Field and Hybrid MR Imaging, University Hospital Essen, University Duisburg-Essen, Essen, Germany; 3 MRI-STaR Magnetic Resonance Institute for Safety GmbH, Technology and Research GmbH, Gelsenkirchen, Germany; 4 MR:Comp GmbH, Testing Services for MR Safety and Compatibility, Gelsenkirchen, Germany

**Keywords:** Artifacts, Numeric simulations, Metallic implants, Magnetic field strength, Orthopedic implants, Human model artifact simulation

## Abstract

**Objective:**

This study presents an extended evaluation of a numerical approach to simulate artifacts of metallic implants in an MR environment.

**Methods:**

The numerical approach is validated by comparing the artifact shape of the simulations and measurements of two metallic orthopedic implants at three different field strengths (1.5 T, 3 T, and 7 T). Furthermore, this study presents three additional use cases of the numerical simulation. The first one shows how numerical simulations can improve the artifact size evaluation according to ASTM F2119. The second use case quantifies the influence of different imaging parameters (TE and bandwidth) on the artifact size. Finally, the third use case shows the potential of performing human model artifact simulations.

**Results:**

The numerical simulation approach shows a dice similarity coefficient of 0.74 between simulated and measured artifact sizes of metallic implants. The alternative artifact size calculation method presented in this study shows that the artifact size of the ASTM-based method is up to 50% smaller for complex shaped implants compared to the numerical-based approach.

**Conclusion:**

In conclusion, the numerical approach could be used in the future to extend MR safety testing according to a revision of the ASTM F2119 standard and for design optimization during the development process of implants.

## Introduction

Over the past decades, the number of patients with passive and active implants who need to be examined with magnetic resonance (MR) imaging has increased [1]. In addition to the safety-related aspects, i.e. the induced heating or the translational forces and torques on the implant, MR imaging artifacts are an important aspect of MR safety assessments [2]. Orthopedic implants, for example, consist of a high proportion of metallic components, and thus may create large MR image artifacts. These are characterized by areas of signal loss and geometric distortions as the implants distort the static magnetic field of the MR scanner and thus prevent accurate spatial encoding of the MR signal [3–5]. This problem occurs especially in the vicinity of these implants and may result in the inability to use MR images for diagnostic purposes. At the same time, orthopedic implants are associated with higher risks of local inflammation [6] and, due to its high soft tissue contrast, MR is the imaging modality of choice for detecting inflammation. For these cases, it is important to know and to predict the exact shape, size, and position of the artifact generated by a specific implant under the chosen imaging sequence and applied scanning parameters. This a priori information allows improved planning of the scanning procedure to achieve the best possible MR image quality. Additionally, it is of interest for implant manufacturers to determine the size and shape of implant-induced MR imaging artifacts for a specific implant under development. The information could be used as an additional design parameter aiming at implants with reduced MR imaging artifact footprints.

Due to the importance of MR-related image artifacts the ASTM F2119 standard was developed to provide a consistent method for testing these artifacts from medical implants. This standard defines basic spin echo and gradient echo sequences with clearly defined sequence parameters to make the artifacts between different test objects (TO) comparable and to get a basic understanding of how large the expected artifacts of medical implants will be [7].

Nevertheless, the results of the ASTM F2119 test method are not transferable to clinical routine MR imaging applications since investigated artifacts are only evaluated under clearly defined conditions in phantom measurements with limited clinical relevance. Additionally, the artifacts according to ASTM F2119 are defined as the maximum distance from the edge of the implant to the fringe of the artifact measured only in the one slice with the largest artifact. This also leads to a lack of information, because there is no information about the TO location inside the artifact, which also depends on the orientation of the implant. Hence, for multi-componential medical implants of complex shape like orthopedic plates and screws, it is impossible to describe these artifacts in a three-dimensional space by only one distance. Furthermore, artifact investigation according to ASTM F2119 includes different test objects and slice orientations, thus constituting a time-intensive measuring procedure that requires a large amount of MR scanning time.

To reduce the aforementioned limitations of the current ASTM method, in this work, a numerical framework for simulating MR image artifacts was extended and used to create an improved artifact investigation technique. This technique is based on a numerical simulation framework which was previously validated with small TOs with a simple geometry [8]. In this current work, more realistic and more complex medical implants will be used to further test and validate our numerical approach. Therefore, simulations and MR measurements of two complex orthopedic implants will be compared, and the influence of different imaging parameters on the resulting MR image artifacts [9] will be quantified with the help of the numerical simulation tool. Furthermore, three use cases of the numerical simulation procedure to improve the accuracy of the investigation of implant-related image artifacts will be presented. One application focuses on an exact placement of the test object inside the MR image artifact and the difference between this method and the method described in the ASTM F2119 standard. Especially for larger artifacts, which appear more pronounced at higher magnetic field strengths, it is usually not possible to accurately determine where the test object is located within the artifact. This limits the exact calculation of the MR image artifact whereas the described technique solves this problem. The second application shows the influence of varying sequence parameters on the resulting size and shape of the artifact, where the third use case extends the numerical simulation to a human body model to show the exact position of the artifact inside the human body and in the context of surrounding tissues.

## Materials and methods

### Performed simulations for validation

**Fig. 1:**
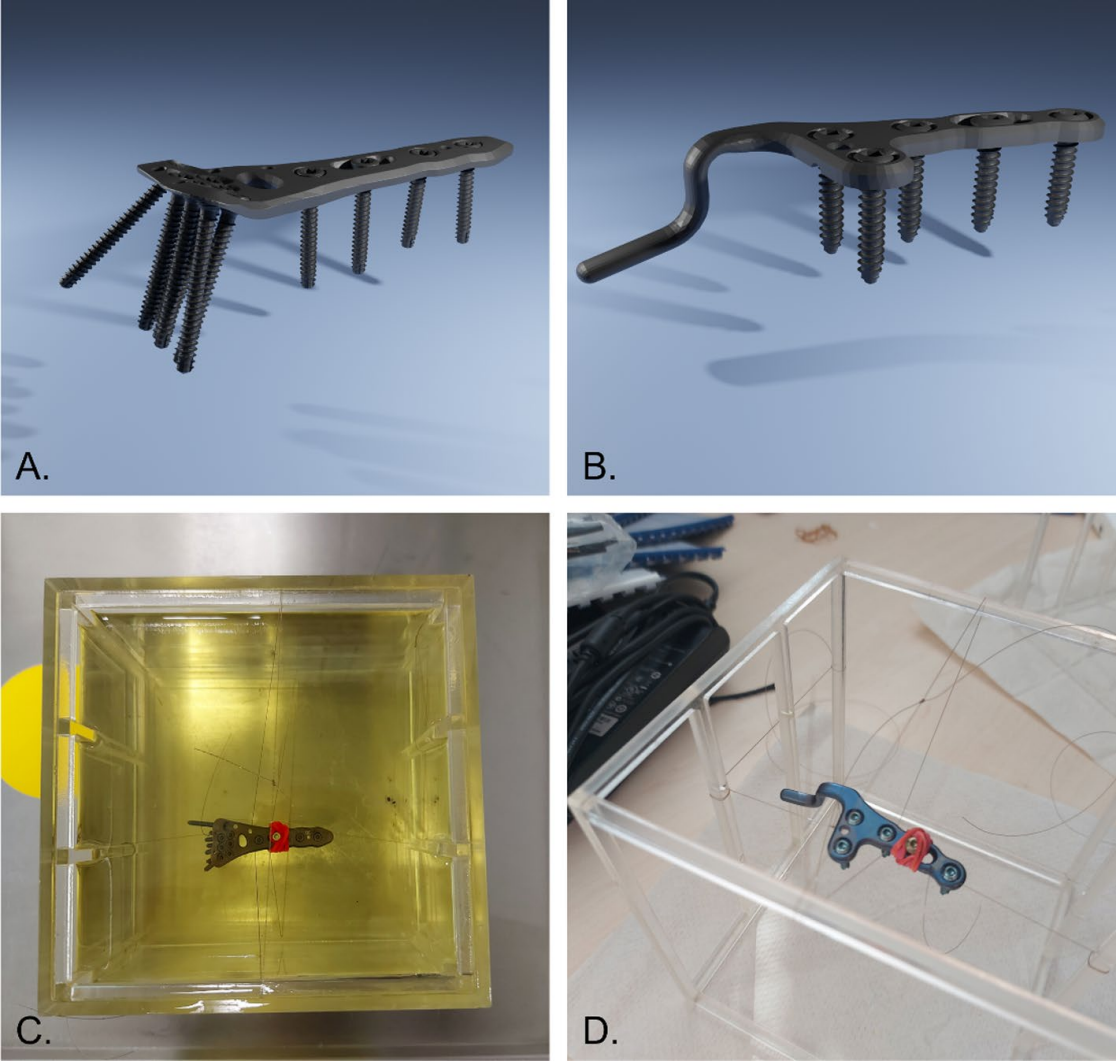
Simulated and measured test objects (Königsee Implantate GmbH, Allendorf, Germany). 3D renderings of CAD models of **A** titanium distal radius plate with eleven angle-stable cortical screws (Ø 2.4 mm, length 20 and 24 mm) and **B** titanium acromioclavicular joint hook plate with five angle-stable cortical screws (Ø 3.5 mm, length 20 mm). Phantom setup for MRI measurements of both implants (**C**, **D**). The implants were fixated with a fishing line in the middle of an oil filled Plexiglas phantom

The MR image artifacts of two medical orthopedic implants were simulated with a resolution of 2 pixel/mm at different magnetic field strengths of 1.5 T, 3 T, and 7 T by using a numerical framework which was developed and validated in a previous study for test objects with simple geometry [8]. Within the present study, two orthopedic implants were available from Königsee Implantate GmbH (Allendorf, Germany), a titanium distal radius plate with eleven angle-stable cortical screws (Ø 2.4 mm, length 20 mm and 24 mm) (Fig. 1A) and a titanium acromioclavicular joint hook plate with five angle-stable cortical screws (Ø 3.5 mm, length 20 mm) (Fig. 1B). Furthermore, geometrically accurate 3-dimensional computer aided design (3D CAD)-data of the two orthopedic implants were provided by the manufacturer. The susceptibility of the two implants was set to 182 ppm according to the material information provided by the manufacturer [1]. For the numerical simulations, the TOs were arranged inside a virtual quadratic phantom (140 × 140×140 mm^3^). The phantom was filled with a virtual medium (T1 = 900 ms, T2 = 50 ms) for all three field strengths. A set of spin echo (TR = 500 ms, TE = 20 ms,) and gradient echo sequences (TR = 500 ms, TE = 15 ms, flip angle = 30°) were simulated for image acquisition with the same parameters as described in ASTM F2119 [7]. Both sequences were acquired with a matrix size of 256 × 256 pixels and a slice thickness of 3 mm. For every sequence, the two TOs in the simulations were sequentially placed inside the phantom in three different orthogonal orientations in relation to the static magnetic field, and for each of the three magnetic field strengths. Furthermore, a set of three orthogonal slice orientations was simulated for each configuration. This led to an overall number of 108 simulations for the validation of the simulation. For the scan configuration of each test object, five slices with a slice thickness of 3 mm each were simulated. In addition to these simulations, reference images that did not contain the TO were simulated for every configuration. These reference images were required to calculate the signal change caused by each TO.

### Simulation of different T1 andT2 times for background medium

It is well known that T1 and T2 times show variation with different magnetic field strengths [10]. The background medium in the simulations was assumed to have the following fixed relaxation times for all three tested field strengths: T1 = 900 ms, T2 = 50 ms. To evaluate the impact of different T1 and T2 times on the resulting artifact sizes, an additional simulation was performed where the T1 and T2 times of the medium were varied each by ± 50% (T1: 450–1250 ms and T2 25–75 ms) while keeping the other parameter constant. Based on these simulations the impact of the T1 and T2 times on the resulting simulated artifact size was evaluated.

### Performed measurements for validation

For the validation of the simulations, MR measurements were performed at 1.5 T, 3 T, and 7 T with the same implants as described before. The two TOs were sequentially placed inside a phantom which was filled with two liters of vegetable oil (*ε* = 0.40, *σ* = 6.6 mS/m) and the TOs were fixed with fishing lines in the middle of the phantom (Fig. 1C/D). Fishing lines were used because they allow a flexible placement of the TO inside the phantom while minimally affecting the imaging procedure. The oil was used for its high imaging contrast and high background signal homogeneity across different magnetic field strengths [11]. The phantom setup for the MR measurements including the two TOs and phantom filling thus resembled the simulated setup using 3D CAD models of the TOs as far as practically possible. Similar to the simulations, the sequence, the TO orientation, and slice orientations were varied for each of the two test objects and for each of the three magnetic field strengths. As for the simulations, this also led to an overall number of 108 MR measurements. The measurements were performed on a 1.5 T MAGNETOM Aera, a 3 T MAGNETOM Skyra, and a 7 T MAGNETOM Terra (all Siemens Healthcare GmbH, Erlangen, Germany). The 1.5 T and 3 T MRI systems were used with a 20-channel radiofrequency (RF) receiving head coil. The 7 T MRI system was used in combination with a 1-channel transmit/32-channel receive head coil (Nova Medical, Wilmington, MA). Based on these measurements, the accuracy of the simulated artifacts was validated.

### Validation procedure

For the validation of the simulations described before, the shape and size of the simulated artifacts were compared pairwise to the according MR phantom measurement of the same configuration. For this purpose, the artifact masks for the simulated and measured artifact images were calculated according to ASTM F2119. Therefore, the pixelwise difference of the image intensity between the MR image containing the test object (${{\varvec{S}}}_{\mathbf{T}\mathbf{O}}$) and the signal of the corresponding reference image (${{\varvec{S}}}_{\mathbf{r}\mathbf{e}\mathbf{f}}$) was calculated and divided by the signal of the corresponding reference image (Eq. 1). This results in a relative signal change (RSC) which is caused by the test object.

1 $$\mathbf{R}\mathbf{S}\mathbf{C}\left({\varvec{x}},{\varvec{y}}\right)=\left|\frac{{{\varvec{S}}}_{\mathbf{T}\mathbf{O}}\left({\varvec{x}},{\varvec{y}}\right)-{{\varvec{S}}}_{\mathbf{r}\mathbf{e}\mathbf{f}}\left({\varvec{x}},{\varvec{y}}\right)}{{{\varvec{S}}}_{\mathbf{r}\mathbf{e}\mathbf{f}}\left({\varvec{x}},{\varvec{y}}\right)}\right|$$

**Fig. 2:**
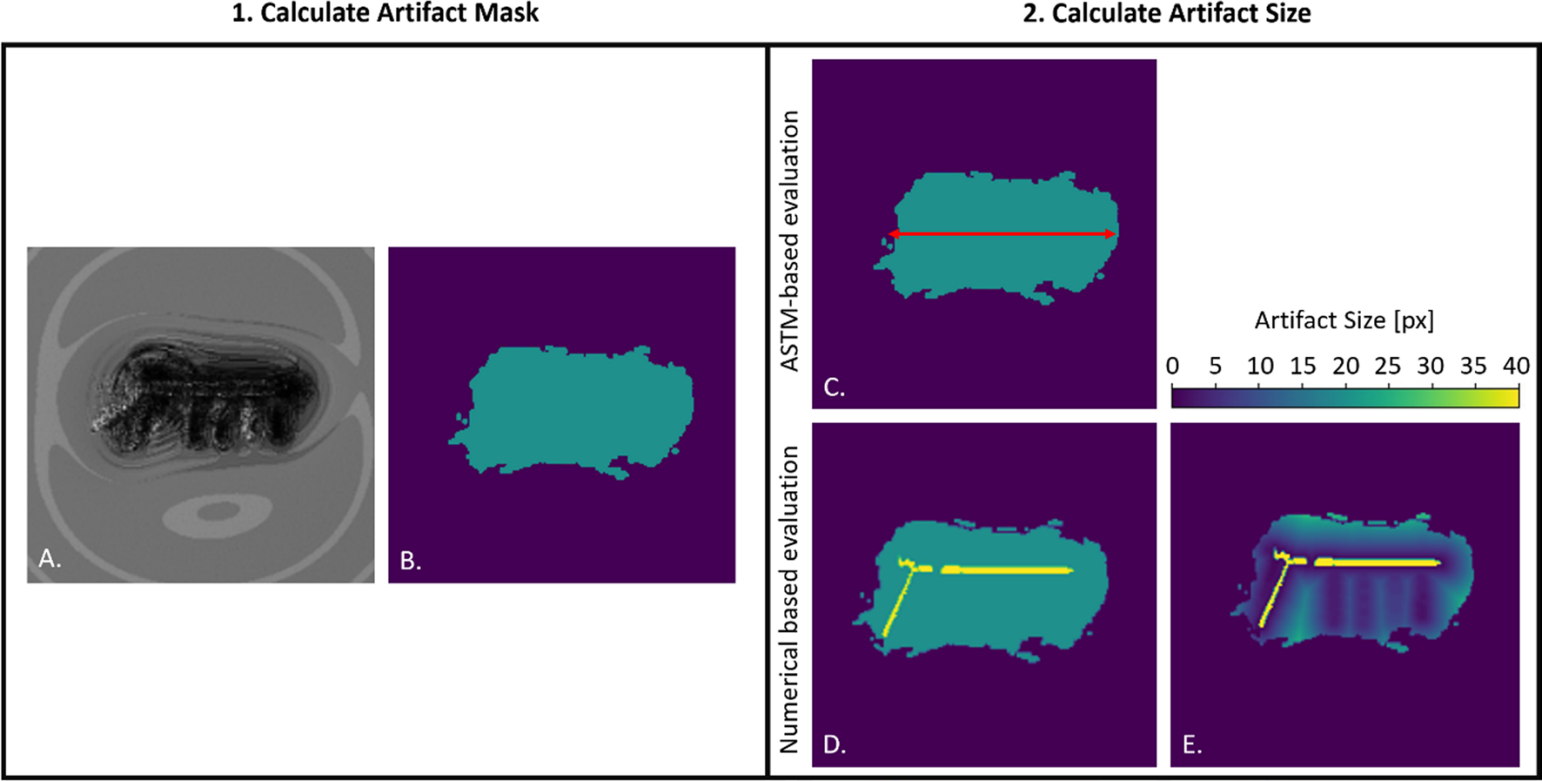
The figure compares the ASTM-based and numerical-based artifact evaluation method of the distal radius plate at 3 T. **A** The simulated magnitude artifact image from which the artifact mask (green) in (**B**) was calculated. **C** The calculation of the artifact size was performed according to ASTM F2119. In **D,** the TO (distal radius plate, yellow) was placed in the artifact mask and in figure **E,** the distance map is shown. Here, the distance from each pixel within the artifact to the test object was calculated. Note that dark colors indicate proximity to the implant while light green colors indicate larger distances to the implant

All pixels with an RSC of more than 30% were described as artifacts and were visualized within an artifact mask as shown in Fig. 2B.

These artifact masks from the simulated and the measured images were superimposed to compare the artifact shape at the same position.

To quantify the agreement between the simulated and the measured artifacts, the areas of the simulated (*A*_sim_) and the measured (*A*_mea_) artifact area were compared using the dice similarity coefficient (DSC) [12]. The DSC was calculated as follows:

2 $$\mathbf{D}\mathbf{S}\mathbf{C}=\frac{2\left|({{\varvec{A}}}_{\mathbf{m}\mathbf{e}\mathbf{a}}\boldsymbol{ }\cap {{\varvec{A}}}_{\mathbf{s}\mathbf{i}\mathbf{m}})\right|}{\left|{{\varvec{A}}}_{\mathbf{m}\mathbf{e}\mathbf{a}}\right|+\boldsymbol{ }\left|{{\varvec{A}}}_{\mathbf{s}\mathbf{i}\mathbf{m}}\right|}.$$

**Fig. 3:**
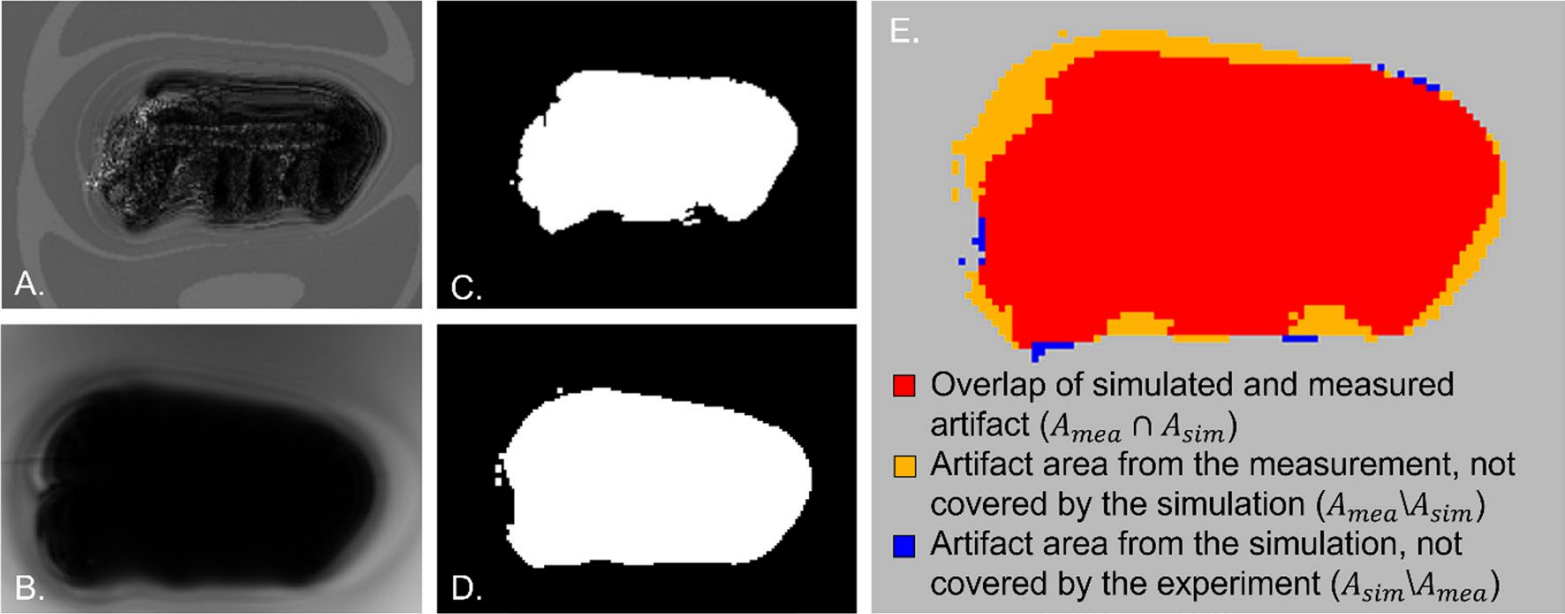
The simulated (**A**) and measured (**B**) magnitude images at 7 T and the corresponding artifact masks **C**, **D** of the distal radius plate are shown. Image **E** exemplarily shows the overlay of the simulated and measured artifact. The dice similarity coefficient (DSC) of this overlay was 0.77

To achieve a better agreement for the superposition of these two artifact masks and to compensate minor positioning errors, the simulation image was shifted in an iterative procedure within the slice by ± 20 pixel in *x* and *y* direction until the highest DSC agreement between the two images was achieved. This final overlay is illustrated as an example in Fig. 3.

Note that in this context higher DSC values represent a better agreement between simulated and measured artifact sizes. As a general acceptance criterion for DSC, a value of larger 0.70 is considered to be a good agreement between simulation and measurement [13, 14].

The DSC was calculated for each of the 108 measurement-simulation pairs and the data were grouped according to the 3 different field strengths (sample size per group *n* = 36) and the 2 sequences (sample size per group *n* = 54). Afterwards, mean, standard deviation, maximum, and minimum of DSC were calculated for each group. 

### Artifact size evaluation approaches

#### ASTM-based evaluation

Based on the artifact masks, the artifact size calculation according to ASTM F2119 was performed. The standard defines an artifact as the distance from the edge of the TO the fringe of the artifact. In cases where the edge of the implant cannot be localized in the image due to large signal voids, an alternative artifact calculation is suggested by the ASTM standard. Here, the total artifact extent (*d*_art_) was measured, and the test object was placed in the center of the artifact. The artifact size was then calculated based on this assumption.

#### Numerical evaluation

The numerical simulation procedure allows an alternative determination of MR imaging artifacts which is visualized in Fig. 2. Therefore, similar to the ASTM-based methods, the artifact masks were calculated first by applying the same criteria as above for the generation of the mask (RSC > 30%) (Fig. 2B). However, in the next step, the TO was not placed in the center of the artifact, but the position information from the simulation input data was used to place the TO at the exact position within the artifact (Fig. 2D).

Based on this placement, the distances to each pixel within the artifact can be calculated and displayed in a distance map (Fig. 2E). Here, both the 3D artifact information and the 3D test object information were used for the distance map calculation, which allows for a cross-layer calculation of the artifact size. This permits a simple visual representation of how large the maximum artifact is, and what the position of the TO relative to the artifact is.

### Extended simulation setup with image parameter variation

In addition to the simulations which were used for the validation procedure, further simulations were performed to quantify the influence of different image parameters on the resulting MR image artifacts. Specifically, the influence of the echo time (TE) and the bandwidth (BW) of a spin echo and gradient echo sequence on the size of artifacts were investigated in more detail. Five different echo times (10 ms, 15 ms, 20 ms, 25 ms, 30 ms) and five different bandwidths (100 Hz/px, 125 Hz/px, 150 Hz/px, 200 Hz/px, 300 Hz/px) were used for both TOs and the entire set of slice and test object orientations. As in the validation simulations, these configurations were simulated for the three magnetic field strengths (1.5 T, 3 T, 7 T).

The changes in artifact size for the specific test configurations were normalized to the lowest value of the parameter (BW: 100 Hz/px and TE: 10 ms) for each test configuration. This eliminates the test configuration specific artifact size that is caused by the orientation of the slice and test object. This comparison allows an evaluation based on the echo time and bandwidth that is independent of the configuration.

### Human model simulation

The numerical approach also allows to simulate artifacts inside a human body model to better display the general appearance of the artifact in relation to the surrounding tissues and anatomy.

Exemplarily, the human model “Duke” (a 34-year-old male) from IT’IS foundation [15] was imported to the simulation with the tissue parameters provided in Table 1. These tissue parameters are assumed to be constant over field strengths. The distal radius plate was placed at the anatomical correct position on the radius bone of the human body model. The entire simulation was performed with a resolution of 2 mm/pixel to achieve an appropriate resolution of the test objects and the human model. The test object was also simulated at three magnetic field strengths (1.5 T, 3 T and 7 T) to show the influence of this parameter.

**Table 1:** The simulated tissue parameters of the human model [23, 24]

Tissue type	T1 [ms]	T2 [ms]	*χ* [ppm]
Air	0	0	0
Bone	365	127	6
Tissue	1300	40	6
Fat	385	120	6

## Results

The results section is divided into four major parts. The first part describes the evaluation of different T1 and T2 values on the artifact size whereas the second section describes the validation of the numerical approach. The third and the fourth part present the potential use cases of the numerical approach in comparison to the ASTM F2119 standard.

### Simulation of different T1 andT2 times for background medium

The T1 and T2 times for the background medium in the final simulations were fixed to T1 = 900 ms, T2 = 50 ms for all three field strengths (1.5 T, 3 T, and 7 T). Variation of the T1 and T2 times by a maximum of ± 50% in simulations did affect the resulting artifact sizes only with very little impact (averaged artifact size change of 1.9%). While the impact on the artifact size was considered neglectable, the variations of T1 and T2 lead to variations of the amplitude of the background signal (brightness). This signal change of the background medium does not affect the calculation of the artifact size, since a reference image with the same background medium was always used in the artifact calculation. Thus, with neglectable impact on artifact size, for the further simulations the T1 and T2 times of medium were fixed to the reported values.

### Validation of the numerical approach

**Fig. 4:**
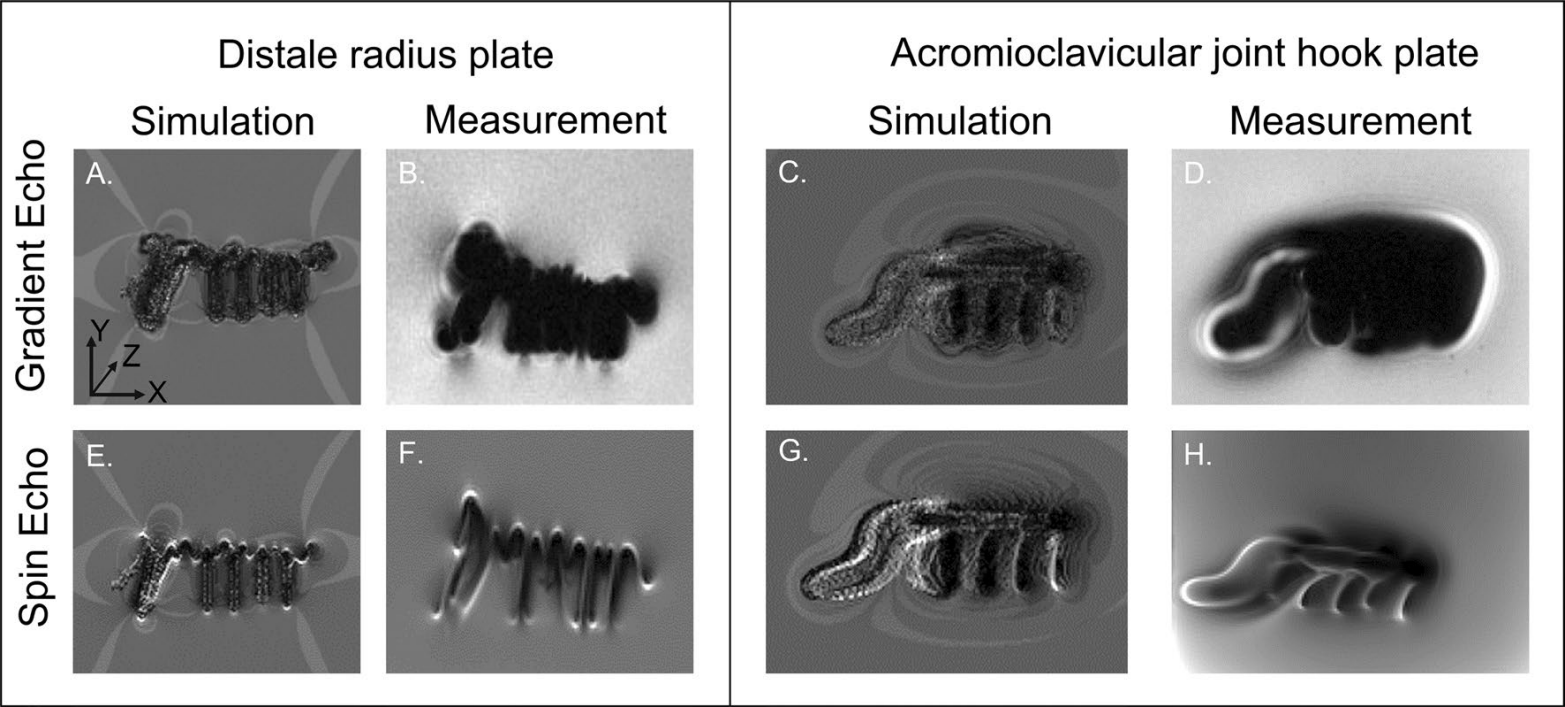
The simulated and measured magnitude images of the distal radius plate (**A**, **B**, **E**, **F**) and the acromioclavicular joint hook plate (**C**, **D**, **G** and **H**) are shown in the center slice of X–Y plane for a gradient echo sequence (**A**–**D**) and for a spin echo sequence (**E**–**H**) at 1.5 T

Figure 4 provides a qualitative comparison of simulations and measurements and shows that the simulation matches the general shape and signal distribution of the measurements for the spin echo and gradient echo images. To quantify the area conformity of this visual comparison, DSCs were used to validate the size and shape of the artifact for each test object configuration.

Across all these different configurations, the average DSC was 0.74 with a standard deviation of ± 0.09. This value is above the acceptance criterion of 0.7 [13, 14], indicating good agreement between the simulation and the measurement. The general DSC values range from 0.47 to 0.92 between the different configurations. Additionality, Table 2 shows the mean, the standard deviation, the maximum, and the minimum values of the DSC separated by sequences and field strengths.

**Table 2:** The dice similarity coefficient (DSC) and the standard deviation of the pixelwise comparison of the simulated and measured artifact images are provided

	Dice similarity coefficient			
	Average	Standard deviation	Maximum	Minimum
1.5 T	0.69	0.09	0.86	0.47
3 T	0.74	0.09	0.91	0.53
7 T	0.79	0.07	0.92	0.63
GRE	0.77	0.08	0.92	0.55
SE	0.70	0.09	0.82	0.47
Overall	0.74	0.09	0.92	0.47

Note that higher DSC values represent higher agreement between simulated and measured artifact sizes

### Comparison of artifact size evaluation approaches

**Fig. 5:**
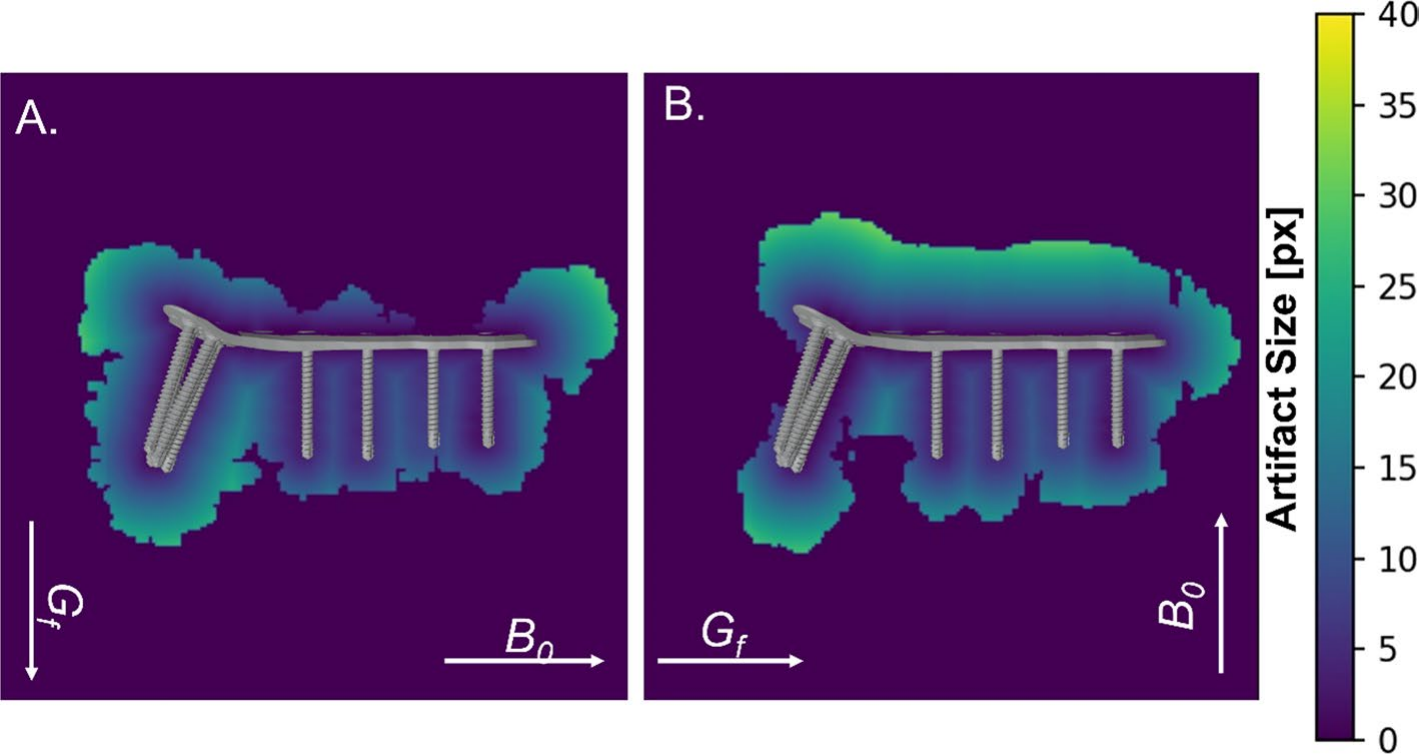
The placement of the TO (distal radius plate) within the distance map at 3 T. **A** Illustration of the artifact with the static magnetic field (*B*_0_) parallel and the frequency-encoding gradient perpendicular (*G*_*f*_) to the plate. **B** Identical TO but this time *B*_0_ oriented perpendicular and G_f_ parallel to the plate. Note how shape and size of artifact change relative to the TO just due to changes in orientation of *B*_0_ and *G*_*f*_

To investigate the difference between the artifact sizes of the ASTM-based and the numerical method, the artifact sizes for the corresponding configurations were compared and the percentage difference between the two methods was determined. Table 3 shows that the difference between the two methods across all test configurations was 33%, which indicates that the numerical method obtains larger artifacts than the ASTM-based one. These differences were more prominent in some configurations, such as the gradient echo sequence of 1.5 T (49%) or the spin echo sequence at 1.5 T (42%). These differences were less prominent for the gradient echo sequences of 7 T and the spin echo sequence of 7 T. The positions of the worst-case artifacts and their distribution can be identified from Fig. 5. Here it can be seen that the orientation of the TO relative to the static magnetic field changed the distribution of the artifact. While in Fig. 5A, the largest artifact was located around the screws, in Fig. 5B, the largest artifact was located at the plate.

**Table 3:** The percentage difference between the ASTM calculation and the numerical artifact size calculation

	GRE	SE
1.5 T	49% ± 15	42% ± 15%
3 T	30% ± 10	30% ± 17%
7 T	19% ± 8	30% ± 16%
Overall	33% ± 14%	

In general, all test configurations based on the ASTM methods led to an underestimation of the artifact size. The averaged underestimation of the artifact size over all test configurations was 33%

It can also be seen in both figures that the TO was not correctly centered in the artifact, especially regarding the frequency-encoding direction. Here, the off-center shift for Fig. 5A was 12 pixels or 7.1 mm and for Fig. 5B, it was 13 pixels or 7.6 mm. This led to artifact sizes for the ASTM-based method which are up to 50% smaller compared to the presented numerical-based approach.

### Extended simulations with image parameters variation

**Fig. 6:**
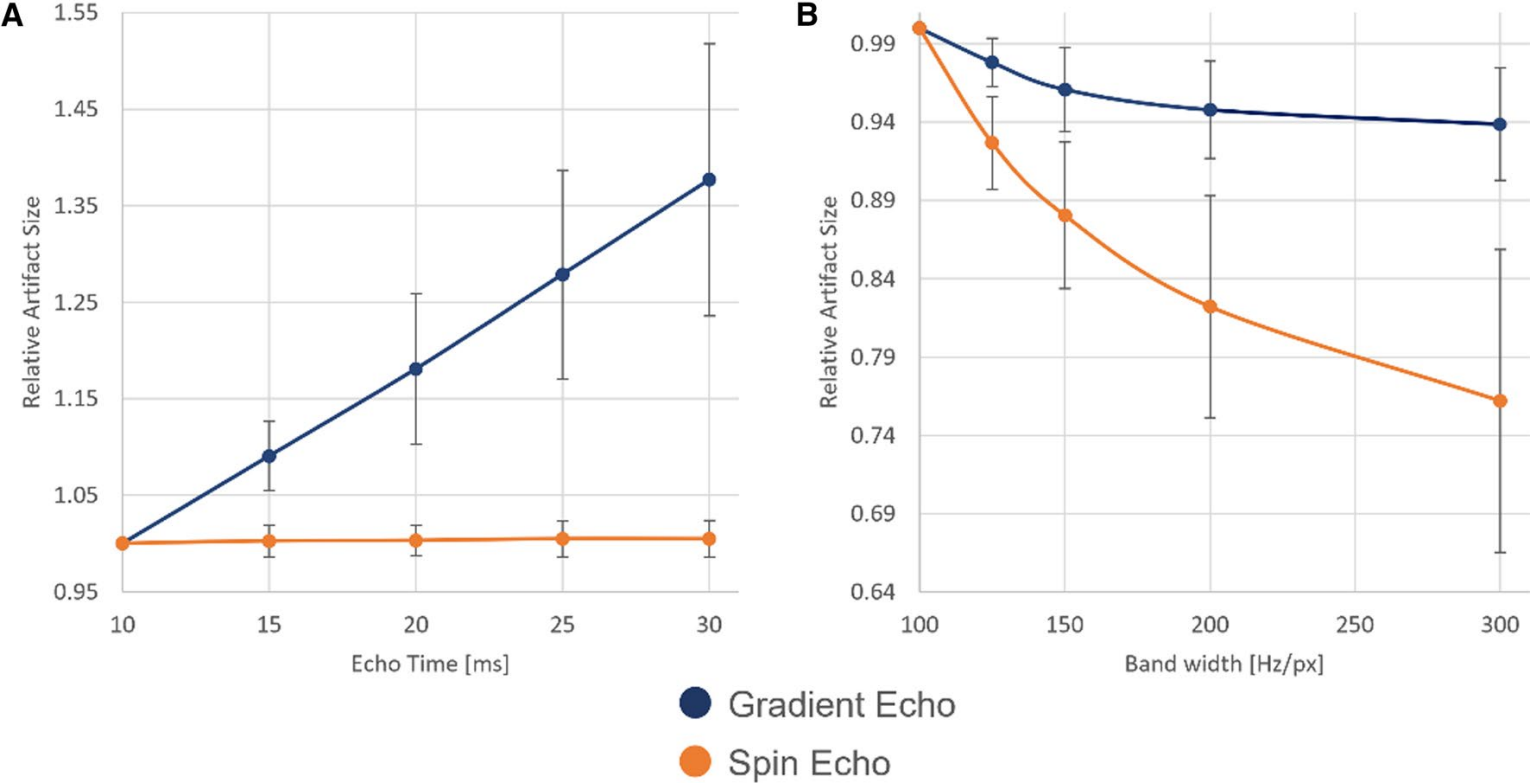
The two graphs show the artifact area as a function of the echo time (**A**) and as a function of the bandwidth (**B**), separated by spin echo (orange) and gradient echo sequence (blue) averaged over 1.5 T, 3 T, and 7 T

The second part of the evaluation focused on additional numerical simulations to show the influence of different scanning parameters on the artifact size. Figure 6 presents the simulated influence of the echo time (TE) and the bandwidth (BW). An increase of the echo time from 10 to 30 ms in the gradient echo sequence led to an increase of the artifact area of more than 50%. In contrast, the spin echo sequences did not show any change in the variation of the standard deviation (Fig. 6A). However, an increase of the bandwidth of 200 Hz/px led to a decrease of the artifact size for both the gradient echo (− 20%) and the spin echo sequence (− 13%) (Fig. 6B).

### Human model simulations

**Fig. 7:**
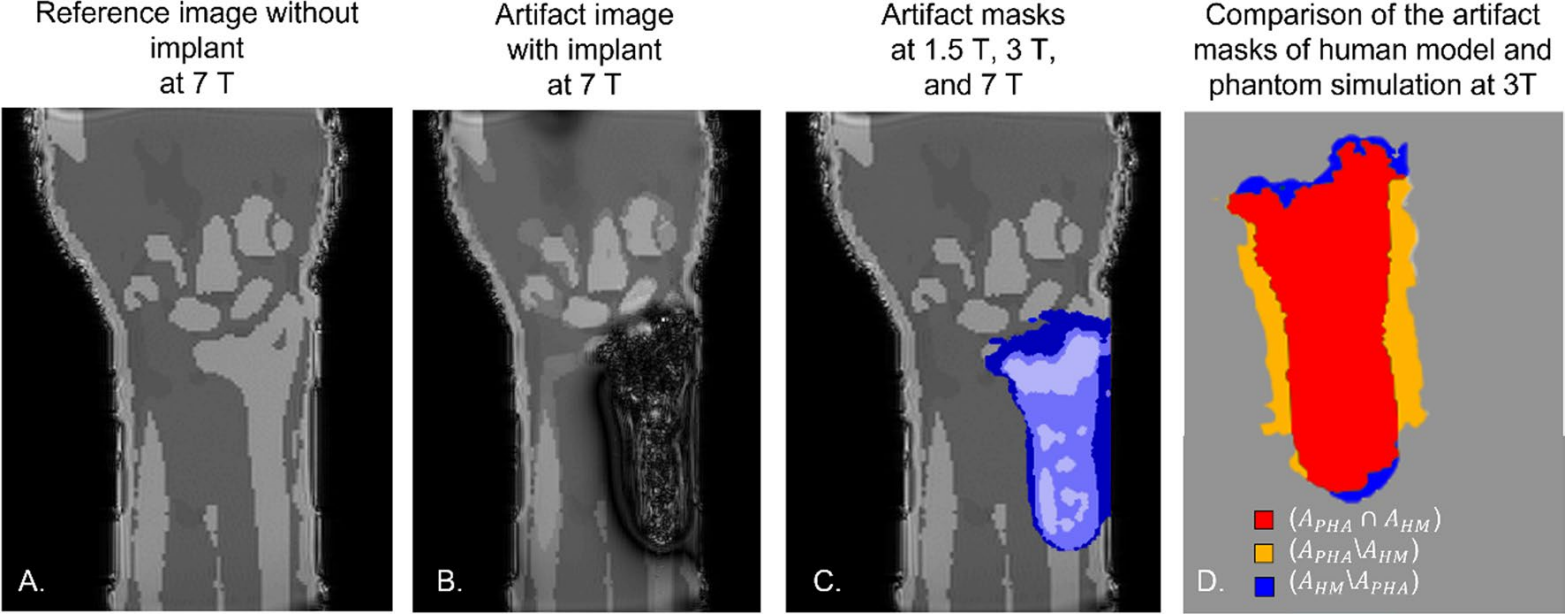
The simulated artifact of the distal radius plate in the context of the hand of the human body model. **A** Reference simulation of the hand without the test object, **B** Simulated artifact at 7 T. **C** Artifact masks at 1.5 T (light blue), 3 T (blue) and 7 T (darker blue). **D** Overlap area of the artifact masks of human model (*A*_HM_) and phantom (*A*_PHA_) simulation (red), artifact area from the phantom simulation, not covered by the human model simulation (yellow), and artifact area from the human model simulation, not covered by the phantom simulation (blue). The DSC between phantom simulation and human model simulation is 0.86

The third use case shows the results of the human model simulations. Figure 7 shows the simulated MR image of a hand at 7 T (Fig. 7A) with the artifacts caused by the distal radius plate in context to the surrounding human tissues (Fig. 7B). Figure 7C shows the artifacts of the distal radius plate at three different field strengths (1.5 T, 3 T, 7 T) in relation to the simulated reference image at 7 T MR image. These simulations provide a more realistic impression of the artifact dimensions and position inside the human body. Figure 7D shows a comparison between the human model simulation and the phantom simulation. The DSC of these two simulations is 0.86. It can also be observed that the artifact of the phantom simulations (red + yellow area) is wider but shorter compared to the human model simulation.

## Discussion

As shown by the averaged DSC (0.74), the artifacts show a high overlap between the simulations and the measurements. Nevertheless, there was a difference between the simulated and the measured MR image artifacts, which may be explained by inaccuracies in the exact placement of the TO and the selection of the correct slice position. Due to the placement of the TO with fishing lines, it was difficult to place the TOs in the phantom with the exact same orientation and at the same position as in the simulation. To reduce this mismatch for the artifacts, the simulations and measurements were overlaid based on the artifact’s masks by an iterative procedure. This procedure, however, only works for the positioning problems within the slice (in plane). The inter-slice positioning error could not be corrected by this procedure. As shown in Fig. 4, the smaller the artifact sizes the more the artifact is shaped like the TO. In contrast the larger artifact from Fig. 3 looks more spherical. These spherical artifacts are less prone to positioning errors when superimposed, and hence provide a higher DSC. However, this could not be avoided since this positioning method was the most practical way to evaluate artifacts without interference. Another potential source of error was the determination of the susceptibility of the TOs. Even small differences between the simulated and the real susceptibility of a TO can lead to a measurable change in artifact size [16, 17]. Despite these potential sources of error, the artifact simulation achieved an overall DSC of 0.74 for these TOs. As mentioned in the material and methods, an DSC > 0.70 provides a good agreement between simulation and measurement and can therefore be used for further investigations.

As shown in the results, the ASTM F2119 standard method yields artifact sizes which are up to 50% smaller compared to the presented approach where the TOs were placed at the exact position. This may be explained by the fact that the artifacts had a larger extension in the frequency-encoding direction [18] and, therefore, the TO was not centered in the artifact [19]. The aforementioned error is relevant in particular for lower field strengths, where this error is not superimposed by intravoxel dephasing [20]. In consequence, it is important to know the exact position of the test object inside the artifact for an accurate artifact determination which could be achieved by visualizing the artifact with the distance map as shown in Fig. 6. This can improve the investigation of the artifact because it is not possible to describe the artifact size of a complex implant by just using a single distance as recommended in the ASTM F2119 standard.

Additionally, the numerical approach allowed an easy adjustment and evaluation of additional imaging parameters (e.g., field strength, TE, or bandwidth) which helped to quantify the influence of different imaging parameters on the resulting artifact sizes. In this study, it was shown that an increase of the TE leads to an increase of the artifact size for the gradient echo images due to an increase of intravoxel phase dispersion before the echo is regenerated [9]. This linear correlation of TE and the artifact size has already been shown in previous studies of Port and Pomper [9]. 

Furthermore, a higher bandwidth leads to a decrease of the artifact size especially for the spin echo sequence. The general artifact size reduction is caused by the lower spatial distortion of the signal for the SE and GRE images. This effect is stronger for the SE compared to the GRE, because here the total artifact is more dominated by the spatial distortion, whereas the GRE artifact is more dominated by the intravoxel dephasing [21]. However, since these studies only performed measurements for a small number of sequences, it was not possible to systematically quantify the influence of these imaging parameters. In this context, it would be advantageous to be able to perform a sequence protocol optimization regarding artifact size reduction via simulations that does not require time-consuming MR measurements. For example, the performed simulations for the different echo times, bandwidths, and field strengths which are shown in Fig. 6, if not simulated, would require a net MR scanning time of about six hours. To create the same set of simulated artifact images, the procedure in its current setup runs for 63 h with multi-core calculation at ten cores. However, these simulations do not require an MR scanner and the simulations, once started, can be completely automated. Furthermore, it can be observed that an increase of the phantom size, or the resolution, increases the simulation time by a factor of *x*^2^. There is a quadratic dependency, because the final simulations are performed on the 2D slice and only the preprocessing (calculation of the off-resonances and the layer profile) is performed using the 3D data set [8]. For this reason, longer simulation times are to be expected for larger test objects. Nevertheless, our research may help developing a guideline that provides quantitative recommendations for optimizing the imaging parameters that lead to a certain change in artifact size for various implants and at various magnetic field strengths.

A slight variation (DSC = 0.86) between the human model and the phantom simulations is shown in Fig. 7. This variation in artifact size is probably not caused by the different material parameters (T1, T2) between the human model and phantom. In this study, it was shown that these parameters have only a limited influence on the artifact size (< 1.9%). In contrast, the tissue–air interface, which is close to the test object in the human model simulation, causes an additional magnetic field distortion [22]. This may be a reason for the different shapes of the two artifacts. Therefore, it may be useful to simulate artifacts under more realistic scenarios, as they will show different artifact shapes.

Numerical simulations are of potential interest for implant manufacturers to assess MR imaging artifact sizes during the development of their products and to design and optimize their implants based on these findings. Additionally, the use of human model simulations (Fig. 7) can be of interest for medical staff, because it allows to see anatomical structures in context with the produced artifact in the specific region of interest. For further improvements of the entire method, the human body model simulations could be additionally run-time optimized so that they can be performed with a variation of imaging parameters.

Concluding, the proposed method could also be used to extend the ASTM-based method, for example by determining the worst-case configuration or by obtaining more detailed information about the artifact by varying sequence parameters.

## Conclusion

A numerical method for the simulation and prediction of MR imaging artifacts generated by realistic and complex orthopedic implants was evaluated and validated. The method provides an accurate and detailed determination of the size and shape of artifacts as a function of field strength, echo time and image bandwidth. The method can be used for MR safety testing according to the ASTM F2119 standard as well as for design optimization during the development process of implants.

## Data Availability

The data that support the findings of this study are available from the corresponding author, Tobias Spronk, upon reasonable request.
